# The Effects of Smoking on Levels of Endothelial Progenitor Cells and Microparticles in the Blood of Healthy Volunteers

**DOI:** 10.1371/journal.pone.0090314

**Published:** 2014-02-28

**Authors:** Fariborz Mobarrez, Lukasz Antoniewicz, Jenny A. Bosson, Jeanette Kuhl, David S. Pisetsky, Magnus Lundbäck

**Affiliations:** 1 Karolinska Institutet, Department of Clinical Sciences, Division of Cardiovascular Medicine, Danderyd Hospital, Stockholm, Sweden; 2 Department of Public Health and Clinical Medicine, Division of Medicine/Respiratory Medicine, Umeå University, Umeå, Sweden; 3 Medical Research Service, Durham VA Hospital, Durham, North Carolina, United States of America; 4 Department of Medicine, Duke University Medical Center, Durham, North Carolina, United States of America; University Hospital Medical Centre, Germany

## Abstract

**Background:**

Cigarette smoking, both active and passive, is one of the leading causes of morbidity and mortality in cardiovascular disease. To assess the impact of brief smoking on the vasculature, we determined levels of circulating endothelial progenitor cells (EPCs) and circulating microparticles (MPs) following the smoking of one cigarette by young, healthy intermittent smokers.

**Materials and Methods:**

12 healthy volunteers were randomized to either smoking or not smoking in a crossover fashion. Blood sampling was performed at baseline, 1, 4 and 24 hours following smoking/not smoking. The numbers of EPCs and MPs were determined by flow cytometry. MPs were measured from platelets, leukocytes and endothelial cells. Moreover, MPs were also labelled with anti-HMGB1 and SYTO 13 to assess the content of nuclear molecules.

**Results:**

Active smoking of one cigarette caused an immediate and significant increase in the numbers of circulating EPCs and MPs of platelet-, endothelial- and leukocyte origin. Levels of MPs containing nuclear molecules were increased, of which the majority were positive for CD41 and CD45 (platelet- and leukocyte origin). CD144 (VE-cadherin) or HMGB1 release did not significantly change during active smoking.

**Conclusion:**

Brief active smoking of one cigarette generated an acute release of EPC and MPs, of which the latter contained nuclear matter. Together, these results demonstrate acute effects of cigarette smoke on endothelial, platelet and leukocyte function as well as injury to the vascular wall.

## Introduction

According to the World Health Organization, cigarette smoking is a major health hazard, and is associated with increased risk of cardiovascular mortality. At the beginning of 2000, more than 1 billion people worldwide were active smokers [1] and it is estimated that 5 million people lose their lives each year due to tobacco use. Tobacco smoking substasntially increases the risk of venous thrombosis and is considered one of the major risk factors for atherothrombotic disease, including ischemic heart disease and stroke [2].

The endothelium covers the innermost layer of the vascular wall and plays a pivotal role in regulating vascular tone, coagulation, fibrinolysis and inflammation [3]. Cigarette smoke has been shown to impair vascular repair, induce platelet activation and cause endothelial damage [4]. *In vitro* data also suggest that cigarette smoke induces endothelial cell activation and/or apoptosis in human aortic endothelial cells [5].

A key cell type in the regulation of vasculature health is the endothelial progenitor cell (EPC). EPCs are mainly derived from the bone marrow and can mediate the differentiation, regeneration and maintenance of endothelial cells in response to vascular injury or vascular neogenesis [6], [7]. EPC levels have been proposed as an important biomarker for endothelial dysfunction. However, the association between levels of EPCs and endothelial dysfunction is still controversial [8]. Several studies have demonstrated that levels of EPCs are inversely correlated to cardiovascular risk factors and that a low EPC count is an independent predictor of cardiovascular events [9], [10].

Another possible marker for endothelial function are microparticles (MPs) of endothelial cell origin. Microparticles, are small membrane-bound particles which are between 0.1–1.0 µm in diameter and circulate in the bloodstream. MPs are released from all types of cells, including leukocytes, platelets and endothelial cells, upon activation or apoptosis of the parent cell. These particles can contain various cytokines, growth factors and proteases and display biological activities that connect them to thrombosis, inflammation and immune responses [11]. Recently, it has been shown that MPs also contain nuclear molecules as shown by SYTO 13 labeling of nucleic acids and binding of antibodies to high mobility group box 1 protein (HMGB1), an important alarmin or danger-associated molecular pattern (DAMP) [12], [13]. SYTO 13, a member of the SYTO dye group, is cell permeable and has a high fluorescent yield when bound to DNA or RNA [14], providing the basis for sensitive detection of structures containing nucleic acids.

Previous studies have shown that chronic smokers display increased levels of circulating endothelial MPs (EMPs) [15]. However, exposure to cigarette smoke has also been shown to have more immediate effects. Heiss et al studied the responses of healthy non-smokers after a 30 minute exposure to second-hand smoke (SHS) and described higher levels of EMPs and EPCs early in the experiment as well as up to 24 hours after SHS exposure [16].

To elucidate further the effects of smoking on the vasculature, we investigated the acute effects of active smoking of one cigarette on the generation of EPCs and MPs in healthy intermittent smokers. These studies indicate rapid changes in these markers, suggesting effects of even one cigarette on a variety of cell types which may contribute to vascular disease.

## Methods

**Figure 1:**
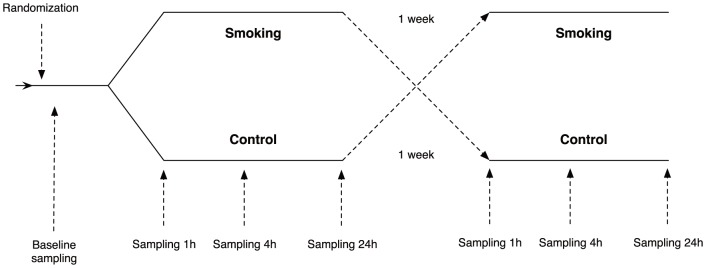
Study design. 12 healthy volunteers were randomized to either smoking or not smoking (control) in a crossover fashion. A time period of at least 1 week separated the experiments. Blood sampling was performed at baseline, 1 h, 4 h and 24 hours.

12 young (mean age 26±5 years, 6 males and 6 females) healthy intermittent/sporadic smokers (max 10 cigarettes per month) were included. Intermittent smokers were recruited due to their ability to completely inhale a cigarette without feeling nausea or sickness. All subjects had a normal health declaration. Exclusion criteria were cardiovascular disease, diabetes mellitus, asthma and/or allergy, respiratory infection within 4 weeks of the study. All subjects smoked one cigarette (John Silver) completely for approximately 5 minutes in a specially prepared temperature-controlled room (22°C), where the exhaled smoke was immediately removed through ventilation. Blood was drawn from an antecubital vein at baseline, 1, 4 and 24 hours after exposure following at least 15 minutes of rest in a semi-supine position (Figure 1). To avoid misinterpretation due to a possible daily variation, blood samplings were also collected without smoke exposure, at least one week apart. Research subjects were required to refrain from alcohol, caffeine and heavy exercise at least 24 hours prior to exposure. Tobacco usage (including Swedish moist snuff) was not permitted within 7 days prior to exposure.

Blinded investigators performed all analysis and written informed consent was obtained from all subjects prior to the study. This study was approved of by the Human Research Ethics Committee at Karolinska Institutet, Stockholm (2012/672-31).

### Blood sampling

Blood was drawn into test tubes containing 1/10 volume sodium citrate. Platelet free plasma was obtained after centrifugation at 2000 *g* for 20 minutes at room temperature (RT) and stored at −70°C until analysis.

### Measurement of endothelial progenitor cells

The numbers of EPCs were measured as CD34^+^ KDR^+^ (KDR: vascular endothelial growth factor receptor 2) double positive cells using a Beckman Gallios flow cytometer (Brea, CA, USA). Briefly, 20 µl of whole blood were incubated with CD34-FITC (Beckman Coulter, Brea, CA, USA) and CD309 (Becton Dickinson, Franklin Lakes, New Jersey, USA). Conjugate isotype-matched immunoglobulin (IgG1-FITC, IgG1-PE) with no reactivity against human antigens was used as a negative control. After 30 minutes of incubation in a dark environment, BD cell-fix was added to fixate the samples. Twenty thousand events of leukocytes were collected (based on classical forward scatter/side scatter (size/granularity) characteristics of blood leukocytes) and results are presented as the number of EPC events.

### Measurement of microparticles

Plasma was thawed and centrifuged at 2000 *g* for 20 minutes at RT. The supernatant was re-centrifuged at 13,000 *g* for 2 minutes at RT. Subsequently, 20 µL of sample was incubated for 20 minutes in the dark with phalloidin-Alexa 660 (Invitrogen, Paisley, UK), lactadherin-FITC (Haematologic Technologies, Vermont, USA), CD41-PE or CD41-APC (Platelet-MP (PMP), Beckman Coulter, Brea, CA, USA), CD45-PC7 (Leukocyte-MP (LMP), Beckman Coulter, Brea, CA, USA), CD14-APC (Monocyte-MP, Beckman Coulter, Brea, CA, USA) and CD144-APC ((EMP), AH diagnostics, Stockholm, Sweden). PMPs were also labeled with CD154-PE (CD40 Ligand, abcam, Cambridge, UK) and EMPs with CD62E-APC (E-selectin, Beckman Coulter, Brea, CA, USA). MPs from platelets and monocytes were also labeled with anti-HMGB1-PE (R&D Systems, Minneapolis, USA). Nuclear staining was performed by labeling samples with SYTO 13 (Invitrogen, Paisley, UK) alone or double stained with anti-HMGB1-PE, CD41-APC, CD144-APC or CD45-PC7. MPs were measured by flow cytometry on a Beckman Gallios instrument. The MP-gate was determined using Megamix beads (BioCytex, Marseille, France), which is a mix of beads with diameters of 0.5 µm, 0.9 µm and 3.0 µm, respectively. MPs were defined as particles less than 1.0 µm in size, negative to phalloidin (in order to exclude cell membrane fragments [17]) and positive to lactadherin or SYTO 13. Conjugate isotype-matched immunoglobulin (IgG1-FITC, IgG1-PE, IgG1-APC and IgG1- PC7) with no reactivity against human antigens was used as a negative control to define the background noise of the cytometric analysis. The absolute number of MPs was calculated by means of the following formula: (MPs counted x standard beads/L)/standard beads counted (FlowCount, Beckman Coulter).

## Statistical analysis

Based on previous published data it was estimated that approximately 12 subjects were needed to detect a 30% difference between treatments with a power of 80% at a p<0.05 significance level (two sided test). Statistical calculations were performed with Graphpad Prism (5.0c) software using a two-factor (time and exposure) analysis of variance with repeated measures. Skewed data were logarithmically transformed prior to statistical analysis. Statistical significance was taken at p<0.05.

## Results

**Figure 2:**
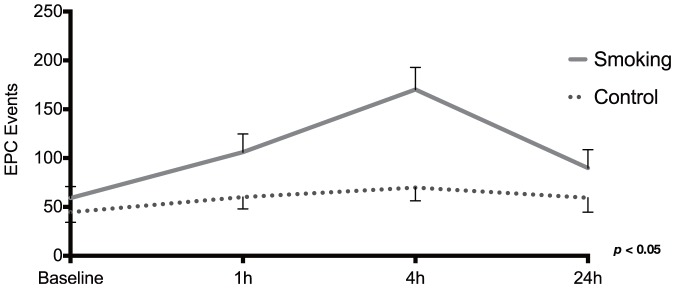
Endothelial progenitor cells during smoking and control. Endothelial progenitor cells were measured as CD34^+^ KDR^+^ double positive cells during smoking and control visit. Data are presented as events of endothelial progenitor cells (mean±SE). Statistical differences (two-factor ANOVA) are indicated in the figure.

Circulating EPCs were measured both during smoking and control visit as CD34^+^ KDR^+^ double positive cells. As shown in Figure 2, active smoking of one cigarette caused a significant increase in the number of circulating EPCs after approximately 1 h and peaked at 4 h (Figure 2).

**Figure 3:**
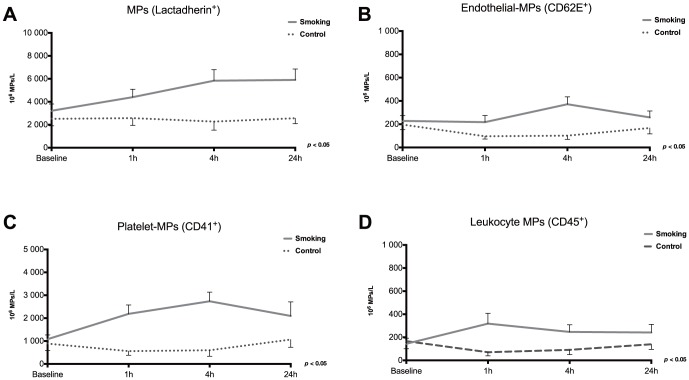
Microparticles during smoking and control. Microparticles were measured by flow cytometry and gated according to size and phosphatidylserine exposure (A) together with cell-specific antigens to determine cellular origin; endothelial-MPs (B), platelet-MPs (C) or leukocyte-MPs (D). Data is presented as mean±SE. Statistical differences (two-factor ANOVA) are indicated for each panel.

We next measured MPs to determine more general effects of smoking as MPs can originate from essentially any cell type that undergoes activation or apoptosis. Results of these experiments are presented in Figure 3 and Table 1. The majority of MP subtypes increased significantly following smoking. MPs that are positive for lactadherin (which binds to exposed phosphatidylserine) and cell-specific MPs (platelets, endothelial and leukocyte origin) were all significantly increased after smoking (Figure 3). Interestingly, only EMPs measured as CD62E (E-selectin) were increased, contrasting with results of staining form CD144 (VE-cadherin) (Figure 3B and Table 1). Further phenotyping also revealed a significant increase in PMPs exposing CD62P and CD154 on their surface (Table 1). HMGB1 exposure did not significantly change in monocyte (CD14) or platelet MPs (CD41) (Table 1).

**Table 1:** Microparticles during smoking and control.

Marker	Exposure	Baseline	1 h	4 h	24 h	*p*
**Lactadherin^+^ CD41^+^ CD62P^+^**	Smoking	268 [51∶789]	335 [193∶1336]	123 [41∶1040]	335 [106∶1021]	*<0.05*
	Control	224 [39∶639]	138 [19∶352]	87 [12∶280]	176 [22∶615]	
**Lactadherin^+^ CD41^+^ CD154^+^**	Smoking	84 [12∶430]	167 [31∶280]	34 [10∶321]	72 [31∶389]	*<0.05*
	Control	87 [7∶135]	41 [1∶77]	19 [5∶176]	80 [7∶203]	
**Lactadherin^+^ CD144^+^**	Smoking	118 [1∶975]	48 [1∶1561]	27 [1∶1129]	121 [29∶1301]	*ns*
	Control	70 [10∶1062]	31 [2∶333]	22 [2∶391]	56 [2∶507]	
**Lactadherin^+^ CD14^+^ HMGB1^+^**	Smoking	80 [10∶536]	331 [12∶1441]	43 [2∶531]	222 [152∶921]	*ns*
	Control	130 [43∶492]	56 [22∶478]	31 [10∶929]	70 [10∶594]	
**Lactadherin^+^ CD41^+^ HMGB1^+^**	Smoking	41 [1∶106]	22 [1∶150]	12 [1∶97]	31 [2∶101]	*ns*
	Control	19 [2∶101]	22 [1∶118]	14 [1∶191]	31 [1∶92]	
**SYTO 13^+^ CD41^+^**	Smoking	138 [36∶536]	331 [12∶1441]	43 [2∶531]	222 [152∶921]	*<0.01*
	Control	130 [43∶492]	56 [22∶478]	31 [10∶929]	70 [10∶594]	
**SYTO 13^+^ CD45^+^**	Smoking	80 [10∶536]	188 [12∶878]	19 [1∶212]	92 [48∶425]	*<0.05*
	Control	63 [14∶176]	22 [5∶169]	5 [1∶145]	22 [2∶241]	
**SYTO 13^+^ CD144^+^**	Smoking	7 [1∶97]	14 [1∶75]	2 [1∶46]	24 [7∶65]	*ns*
	Control	17 [2∶58]	5 [1∶46]	2 [1∶51]	5 [1∶43]	

Microparticles were measured by flow cytometry and gated according to size and phosphatidylserine exposure (lactadherin binding) together with cell-specific antigens CD41 (platelet origin), CD144 (endothelial origin) or CD14 (monocyte origin). Platelet microparticles were also investigated for P-selectin (CD41+CD62P), and CD40 ligand (CD41+CD154) exposure. Furthermore, both platelet (CD41) and monocyte (CD14) microparticles were labeled with anti-HMGB1. To asses nuclear content and origin, microparticles were labeled with SYTO 13 and cell-specific antibodies; CD41 (platelet), CD45 (leukocyte) and CD144 (endothelial). Data is presented as median and interquartiles [25∶75]. ns: non significant.

**Figure 4:**
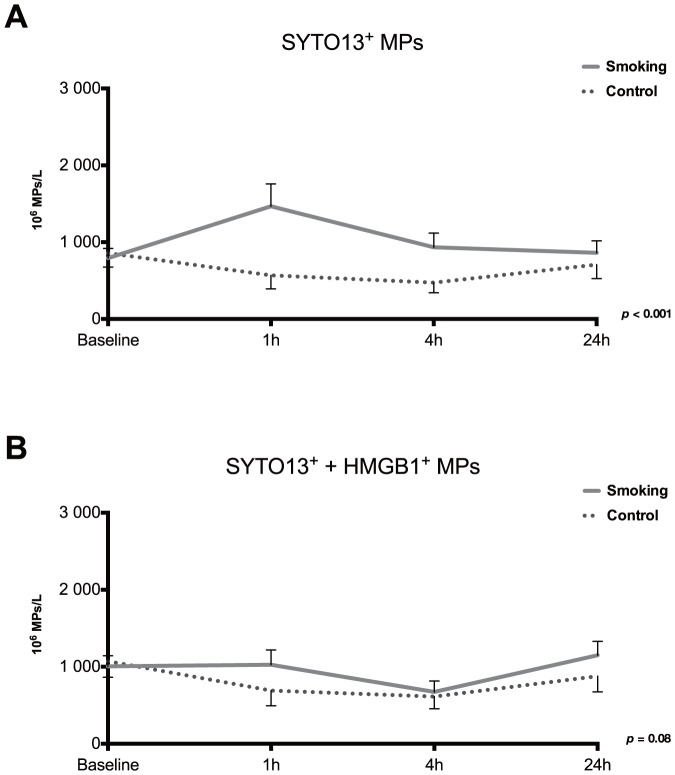
Nuclear molecule content of microparticles after smoking and control. Microparticles were measured by flow cytometry and gated according to size and SYTO 13 binding alone (A) or together with HMGB1 exposure (B). Data is presented as mean±SE. Statistical differences (two-factor ANOVA) are indicated for each panel.

Nuclear content was measured in MPs by assessing SYTO 13 binding (Figure 4A). Data showed a significant increase in all MPs containing nuclear molecules, where the majority were also positive for CD41 and CD45 (platelet and leukocyte origin, Table 1). HMGB1 exposure in SYTO 13^+^ MP population was not significantly changed although it showed some tendencies towards higher levels during smoking (Figure 4B). SYTO 13 binding together with CD144 showed no significant changes (Table 1).

## Discussion

The present study provides new insights into the effect of smoking on the function of the endothelium as blood cell activation, demonstrating that active smoking of one cigarette causes an immediate increase in the number of circulating EPCs as well as MPs of leukocyte, platelet and endothelial origin. Furthermore, active smoking caused an increase in levels of circulating MPs containing nuclear molecules. Taken together, these results show that smoking as little as just one single cigarette for a short period of time can cause MP-release as well as EPC mobilization. These findings suggest immediate systemic events that can impact on vascular integrity and could promote events important in atherosclerosis.

Following smoking of one cigarette, circulating EPCs measured as CD34^+^ and KDR^+^ were significantly increased (Figure 2). In this study, EPCs were identified as CD34^+^ and KDR^+^ (also known as vascular endothelial growth factor receptor 2). At present, a standardized flow cytometry protocol for EPC measurement is not available [18]. While we could have used additional markers such as CD133 to identify EPCs, our data still show a significant difference between smoking and control visits. This rapid EPC response may seem surprising in terms of known mechanisms for the expression of these cells in the blood. As shown in studies of diabetic mice, EPCs are recruited from the bone marrow in a fashion dependent on endothelial nitric oxide synthase [19]. However, the time frame for this response in the present study suggests that bone marrow recruitment cannot account for the quick increase in EPCs demonstrated less than 1 h after smoking. Animal studies have also demonstrated the presence of EPCs dwelling in the vascular wall as well as in the bronchial wash of mice [20]. Furthermore, in a study by Imaoka et al, EPCs have been found in the human lung in patients with asthma [21]. The initial rapid response after active smoking may therefore be explained by a release of EPCs from the bronchial tree and/or the vascular wall rather than bone marrow recruitment.

Cigarette smoke causes increased oxidative stress and reduced levels of nitric oxide (NO) in plasma [22]. Chronic smokers have reduced EPC levels, most likely due to a decrease in NO-bioavailability and resulting reduction in bone marrow recruitment of EPCs [23]. There is an association between low levels of EPCs and conditions characterized by chronic inflammation such as rheumatoid arthritis, systemic lupus erythematosus and ulcerative colitis. Furthermore, circulating EPC-levels are reduced with progression of the disease, implying a gradual depletion of EPCs in the bone marrow [24], [25]. In addition to inflammation, these inflammatory diseases are all associated with vascular injury as well as vascular neogenesis [25].

Until recently, it was not clear whether EPCs were released as a part of a systemic inflammatory response or vascular injury. In a study presented by Padfield et al, healthy individuals were exposed to salmonella typhus vaccination; following this exposure, EPC-levels remained unaffected.[26]. Therefore, the authors concluded that EPC mobilization was due to vascular injury rather than systemic inflammation. Hence, it is not unexpected to see an increased number of EPCs following cigarette smoking. This response may be interpreted as a sign of acute vascular injury, which in the long run leads to a depletion of EPC-storages and reduced capability of vascular repair [12].

Results of the present study showed that smoking increased levels of MPs from platelets, endothelial cells and leukocytes, which most likely is due to cell activation in response to cigarette smoke. We observed an increase of platelet-MPs exposing CD40 Ligand (CD154) and CD62P. CD40L is a co-stimulatory molecule expressed mainly on T cells, although it is also present and functionally active on platelets [27]. CD40L on platelets can activate endothelial cells to secrete chemokines, thereby initiating an inflammatory response [28]. CD62P plays an important role in the recruitment of leukocytes by binding to its ligand, P-selectin glycoprotein ligand-1 and can also be expressed on platelets as well as on endothelial cells [29].

The increase in MPs expressing CD45 (leukocyte origin) confirms results in a previous in vitro study by Li et al, in which human monocytes/macrophages exposed to tobacco smoke extract, generated pro-coagulant MPs [30]. Also, type 2 diabetic smokers showed increase in circulating tissue factor after smoking two cigaretts, thus leading to an increase in pro-coagulant state [31]. Circulating tissue factor is believed to be bound to circulating microparticles [32]. As MPs are considered pro-coagulant [33], due to exposed phosphatidylserine or tissue factor, an increase of MPs after one smoked cigarette may offer a mechanistic explanation for the association between cigarette smoke and thrombotic disease. Indeed, smoking is known to be a major risk factor for cardiovascular disease [34] and numerous studies have demonstrated increased levels of MPs in diseases such as acute coronary syndrome and ischemic stroke [35].

EMPs were measured as either CD62E (E-selectin) or CD144 (VE-cadherin)-positive EMPs, of which only CD62E^+^ MPs increased (Figure 3 and Table 1). CD144 is a large molecule located in tight junctions between endothelial cells [36]. Consequently, more damage may be required to the endothelial cells until CD144^+^ MPs are released. During exposure to second-hand smoke, both CD62E^+^ and CD144^+^ MPs were increased [16]. Whilst the exposure of second hand smoke was 30 minutes, our volunteers only smoked one cigarette for approximately 5 minutes; this difference could influence the findings in the various studies. Results also showed that the amount of HMGB1 exposed on monocytes or platelet MPs was not altered during active smoking. Extracellular HMGB1 is a prototype alarmin that can display pro-inflammatory activity depending on its origin during activation and apoptosis and has previously been found on microparticles [13], [37]–[38]. Interestingly, it has been shown that nicotine can suppress HMGB1 release from macrophages through a nicotinic receptor dependent pathway [39]. Since we only measured exposed HMGB1 on MPs by flow cytometry, we cannot be certain if the nicotine in cigarette smoke has reduced or “masked” a potential effect on HMGB1 levels that may result from other ingredients in smoke or their actions. Soluble HMGB1 can also be measured by conventional ELISA. However, we have previously shown that measurement of MP-bound HMGB1 measured by flow cytometry is more sensitive than ELISA measurement [13].

In addition to a more conventional MP measurement, we also labelled MPs with SYTO 13 together with cell-specific markers or anti-HMGB1 (Figure 4 and Table 1). As previously shown [12], the binding of SYTO 13 by MPs generated from apoptotic cells *in vitro* reflects the presence of both RNA and DNA that likely occurs during translocation of nuclear molecules into particles. Also, LPS, which is known to induce apoptosis, can lead to the release of MPs containing DNA/RNA [13]. In this study levels of all MPs positive for SYTO 13 were significantly altered (Figure 4A) and further phenotyping revealed that the majority of MPs were from platelets and leukocytes (Table 1). Interestingly, platelets lack a nucleus but still contain mitochondrial DNA and mRNA, as well as HMGB1 [40]–[41]. It is tempting to speculate that these MPs containing nuclear matter were released due to apoptosis, although cell death would be expected to take more time. Nevertheless, an increase in these MPs was noted after only one smoked cigarette. Moreover, MPs measured with SYTO 13 and CD144 as well as SYTO13 and HMGB1 did not significantly change, most likely due to reasons described above.

In summary, smoking as little as one cigarette for a brief period of time can cause an early release of EPCs, as well as MPs containing nuclear molecules, which can have pro-inflammatory activity into the circulation. Because of the origin of MPs, their presence in the blood after smoking suggests important systemic effects in terms of inflammation, impaired endothelial function and vascular injury and repair. Future studies are necessary in order to fully determine the role of biomarkers such as EPCs and MPs, as markers to elucidate both immediate and long-term effects of smoking on the endothelium and blood cells.
